# Galectin-3 not Galectin-9 as a candidate prognosis marker for hepatocellular carcinoma

**DOI:** 10.7717/peerj.9949

**Published:** 2020-09-16

**Authors:** Fei Kong, Meishan Jin, Donghui Cao, Zhifang Jia, Yawen Liu, Jing Jiang

**Affiliations:** 1 Division of Clinical Research, the First Hospital of Jilin University, Changchun, China; 2 Department of Hepatology, the First Hospital of Jilin University, Changchun, China; 3 Department of Epidemiology and Biostatistics, School of Public Health, Jilin University, Changchun, China; 4 Department of Pathology, the First Hospital of Jilin University, Changchun, China

**Keywords:** Hepatocellular carcinoma, Galectin-3, Galectin-9, Prognosis

## Abstract

**Background:**

Galectins (Gal) are a family of protein that bind to the *β*-galactoside of glycoproteins. It modulates a variety of biological functions, such as tumor growth, angiogenesis and tumor metastasis. A series of experimental and clinical evidences have been reported to support a correlation between galectin expressions and neoplastic transformation, progression and prognosis. The objective of this study was to estimate the expression of Gal-3 and Gal-9 in order to evaluate their relation to hepatocellular carcinoma (HCC) -related clinical features and their prognostic values.

**Methods:**

We evaluated Gal-3 and Gal-9 expression in 247 HCC patients by a tissue microarray immunohistochemistry method, then analyzed the relationship between expression levels of Gal-3 and Gal-9 protein and tumor parameters or clinical outcomes.

**Results:**

The Gal-3 expression was significantly higher in tumor tissues compared with adjacent non-tumor tissues (*P* < 0.001), while no significant differences of Gal-9 was detected (*P* = 0.222). A higher Gal-3 expression was significantly associated with lymph-vascular invasion (*P* = 0.049), poor histological differentiation (*P* = 0.016), and no cirrhosis (*P* = 0.040). In contrast, a lower Gal-9 expression was related to lymph-vascular invasion (*P* = 0.012) and poor histological differentiation (*P* = 0.002). Survival analysis showed that patients with higher Gal-3 expression had worse overall survival (*P* = 0.012) , however no correlation was found between Gal-9 expression and survival (*P* = 0.185). Multivariate analysis showed that multiple tumor (HR = 1.94, 95% CI [1.36–2.78]), tumor size ≥ 5 cm (HR = 1.51, 95% CI [1.07–2.12]), Lymph-vascular invasion (HR = 1.45, 95% CI [1.00–2.10]) and Gal-3 expression (HR = 1.57, 95% CI [1.06–2.33]) were independent influencing factors of prognosis in patients with hepatocellular carcinoma.

**Conclusion:**

Gal-3 was expected to serve as a novel prognostic marker of hepatocellular carcinoma, while Gal-9 expression was only related to tumor progression.

## Introduction

Hepatocellular carcinoma (HCC), which accounts 75∼85% of primary liver cancer, remains the sixth common cancer worldwide and the fourth most cause of cancer mortality (Bray et al., 2018). In China, over 422,000 people die every year as the result of liver tumors (Chen et al., 2016). Surgical resection is the most common treatment method for early-stage HCC, local ablation, transcatheter tumour treatment, chemotherapy, multikinase inhibitor and immunotherapy are options for patients who are unsuitable for surgical resection (Johnston & Khakoo, 2019). However, the outcome and prognosis of HCC are disappointing, about 70% subjects develop recurrence within 5 years after resection (Tabrizian et al., 2015). The five-year survival is poor compared with other gastrointestinal malignant tumors (Bosetti, Turati & La Vecchia, 2014). In China, the five-year survival rate is about 37% after surgical resection (Yang et al., 2015b). However, for the low resectable rate at the time of diagnosis the five-year survival rate is only 12.1% in all liver tumors patients (Zeng et al., 2018). Some factors can predict the outcomes and prognosis of HCC, however, existed conventional prognostic indicators are poor at predicting the prognosis of HCC  (Wu et al., 2019; Sapisochin & Bruix, 2017). Therefore, discovering new marker for predicting the prognosis of HCC after surgical resection is crucial to improve its management and long-term survival.

As a family of animal lectins, galectins (Gal) bind β-galactosides through conserved sequence elements of the carbohydrate recognition domain (CRD). Galectins are involved in the pathogenesis of HCC. Gal-1, Gal-3 and Gal-4 are up-regulated in HCC cells, whereas Gal-8 and Gal-9 are down-regulated in tumor hepatocytes  (Wu et al., 2012; Jiang et al., 2014; Cai et al., 2014; Zhang et al., 2012). This abnormal expression relates to tumor growth, tumor cell migration and invasion, tumor metastasis, and poor prognosis. Gal-3 and Gal-9 also play key roles in inflammation- and fibrosis-related liver pathologies (Bacigalupo et al., 2013).

Galectin-3 is a multifunctional member of galectin family. It has been reported that Gal-3 is over-expressed in several cancers, including breast cancer, cervical cancer, gastric cancer, colon cancer and renal cell cancer (Ebrahim et al., 2014). Higher expression of Gal-3 can suppress tumor cell apoptosis (Nakahara, Oka & Raz, 2005), facilitate tumor metastasis by modulating innate anti-tumor immunity (Radosavljevic et al., 2012) and stimulate tumor capillary tube formation  (Nangia-Makker et al., 2000). In HCC, related research reported that expression of Gal-3 is associated with vascular invasion and histological differentiation, up-regulated expression of Gal-3 was closely related to a poor prognosis (Matsuda et al., 2008). However Gal-3 expression is not significantly associated with clinicopathological parameters in the study of Jiang et al. (2014).

Galectin-9 is a new member of the galectin protein family (Jiang et al., 2013). The role of Gal-9 in cancer is complicated. In breast cancer, it can induce tumor cell aggregation and adhesion, and high Gal-9 expression is closely related to reduced metastasis and low recurrence (Irie et al., 2005; Yamauchi et al., 2006). Similar findings were also observed in malignant melanoma and HCC (Zhang et al., 2012; Kageshita et al., 2002). However in clear-cell renal cell carcinoma, high expression of Gal-9 is associated with poor survival and early recurrence (Fu et al., 2015). Paradoxically, Gal-9 is a ligand of T cell immunoglobulin and mucin-domain containing-3 (TIM-3), combining with TIM-3, Gal-9 triggers the termination of T-cell mediated immunity (Li et al., 2012).

Conflicting results exist on the analysis of relationship between clinicopathological parameters and Gal-3 expression (Jiang et al., 2014; Matsuda et al., 2008) and the limited reports of Gal-9 in HCC in previous literatures. Hence, the function of Galectin-3 and Galectin-9 and their correlation with tumor prognosis remain largely unknown. To evaluate the role of the expression of Gal-3 and Gal-9 during HCC occurrence and their prognostic values, we examined the Gal-3 and Gal-9 protein expression in HCC tumor tissue and paired non-tumor tissue, then explored the association of the protein expression with patient clinical characteristics and prognosis in this study.

## Material and Methods

### Study population

Between October 2008 and May 2013, a total of 247 patients including 195 men and 52 women with HCC who underwent surgery at the First Hospital of Jilin University were enrolled in this study. No patients received chemotherapy or radiotherapy before the surgical operation. Two independent pathologists confirmed the diagnosis of HCC and evaluated immunohistochemical findings. Paired adjacent normal samples were collected from 110 patients for comparison. Patients age range from 23 to 83 years, with a mean age of 54.3 years.

### Ethics statement

The study protocol was approved by the Ethics Committee of the First Hospital of Jilin University (18YY228-001). Written informed consent was obtained from all of the patients.

### Follow-up and outcomes collection

All the subjects underwent follow-up by telephone calls in three months, six months, and one year after the surgical resection and every one year later until death or the last scheduled follow-up. The subjects who were lost to follow-up at the first time of telephone interview, or died of compliments of the surgical operation in the perioperative period were excluded from the survival analysis. The duration from the date of surgical operation to the date of death or the date of the last successful interview was defined as the survival time. The last interview of this study was in May 2019.

### Immunohistochemistry

Briefly, after routine dewaxing, the sections (4 um in width) from the tissue microarry (TMA) blocks were boiled in the 10mmol citrate buffer for 2.5min in a pressure cooker for epitope retrieval. After blocking endogenous peroxidase activity with 3% H_2_O_2_, all sections were incubated with anti-human galectin-9 polyclonal antibody (1:250 diluted, ab69630, Abcam, UK) and galectin-3 monoclonal antibody (1:100 diluted, ZA-0534, Zhongshan jinqiao, China) for 2.5 h at 37 °C. The slides were further treated with horseradish peroxidase-labeled secondary antibody for 15 min at room temperature (MXB, China). The slides were visualized using 3, 3-Diaminobenzidine (DAB) and were counterstained with hematoxylin. For negative controls, tumor slides were treated with the IgG isotypes from rabbit (normal rabbit IgG, sc-2027, Santacruz, USA) to replacement of primary antibody. All negative controls demonstrated negligible background stain.

Two independent pathologists who were blinded to clinical data and outcome evaluated the stained slides. The expression of Gal-3 and Gal-9 were assessed by doing an H-sore, which combines staining intensity and percentages of cells stained (McCarty Jr et al., 1985). The H-score (range of 0–300) was calculated using the following equation: H-score = i1 × P1 + i2 × P2 + i3 × P3. i means the intensity of staining (no staining = i0, weak staining = i1, moderate staining = i2 and strong staining = i3) and P represents percentages of stained cells within intensities varying from 0 to 100. The H-score = 0 is defined as a complete negative staining. The discrepancies of stained cells were resolved by calculating the means of the scores, and the discrepancies of staining intensity were discussed by the two pathologists to reach a consensus.

### Statistical analysis

The H-score of Gal-9 and Gal-3 expression are presented as (interquartile ranges) and categorical variables were expressed as numbers and frequencies (%). The Wilcoxon matched-pairs signed-rank test was used when comparing the expression of Gal-3 and Gal-9 in tumor tissue and adjacent non-tumor tissue (Table 1). The Mann–Whitney test was used to evaluate the correlation between clinicopathological characters and expressions of Gal-3 and Gal-9 in tumor tissue (Table 2). Optimal cut-off values of Gal-3 and Gal-9 were calculated by time-dependent receiver operating characteristic (ROC) curves in the R language for survival analysis. The overall survival rate was estimated using Kaplan–Meier method and compared by the log-rank test. The Cox proportional hazards model was performed to assess factors potentially associated with prognosis of patients with HCC (Table 3). Variables with *P* values below or equal to 0.10 in univariate analysis were included in the multivariate model. Hazard ratio (HR) and corresponding 95% confidence intervals (95% CI) were used to determine the strength of statistical associations. A two-sided *P*-value below or equal to 0.05 was considered significant. Statistical analyses were performed using SPSS software package 18.0 (SPSS Inc. USA).

**Table 1:** The expressions of Galectin-3 and Galectin-9 in tumor tissue and adjacent non-tumor tissue.

Group	*N*	Expression of Galectin-3		Median H-score (quartile)	*P*	Expression of Galectin-9		Median H-score (quartile)	*P*
		0–120	121–300			0–100	101–300		
Tumor tissue	110	77 (70.0%)	33 (30.0%)	0(0–180)	<0.001	68(61.8%)	42(38.2%)	90(10–180)	0.222
Adjacent non-tumor tissue	110	100(90.9%)	10(9.1%)	0(0–0)		59(53.6%)	51(36.4%)	90(90–180)	

**Table 2:** Correlation between clinicopathological characters and expressions of Galectin-3 and Galectin-9.

Character	*n*	Expression of Galectin-3		Median H-score (quartile)	*P*	Expression of Galectin-9		Median H-score (quartile)	*P*
		0–120	121–300			0–100	101–300		
Gender									
Male	195	157 (80.5%)	38 (19.5%)	0(0–80)	0.390	110 (56.4%)	85 (43.6%)	90(40–180)	0.984
Female	52	44 (84.6%)	8 (15.4%)	0(0–90)		28 (53.8%)	24 (46.2%)	90(30–180)	
Age									
≤55ys	127	100 (78.7%)	27 (21.3%)	0(0–120)	0.110	74 (58.3%)	53 (41.7%)	90(20–180)	0.643
>55ys	120	101 (84.2%)	19 (15.8%)	0(0–60)		64 (53.3%)	56 (46.7%)	90(60–180)	
HBV/HCV									
HBV positive	158	123 (77.8%)	35 (22.2%)	0(0–120)	0.052	90 (57.0%)	68 (43.0%)	90(30–180)	0.817
HCV positive	45	41 (91.1%)	4 (8.9%)	0(0–0)		25 (55.6%)	20 (44.4%)	90(40–180)	
Negative	44	37 (84.1%)	7 (15.9%)	0(0–60)		23 (52.3%)	21 (47.7%)	90(40–180)	
Number of tumor									
Single	183	151 (82.5%)	32 (17.5%)	0(0–80)	0.061	99 (54.1%)	84 (45.9%)	90(50–180)	0.561
Multiple	64	50 (78.1%)	14 (21.9%)	7.5(0-112.5)		39 (60.9%)	25 (39.1%)	90(5–180)	
Tumor size									
≤5 cm	137	117 (85.4%)	20 (14.6%)	0(0–80)	0.334	99 (54.1%)	84 (45.9%)	90(60–180)	0.427
>5 cm	110	84 (74.6%)	26 (23.4%)	0(0–120)		39 (60.9%)	25 (39.1%)	90(0–180)	
Differentiation									
Poor	75	59 (78.7%)	16 (21.3%)	15(0–100)	0.016	49 (65.3%)	26 (37.4%)	80(0–180)	0.002
Moderate/well	172	142 (82.6%)	30 (17.4%)	0(0–75)		89 (51.7%)	83 (48.3%)	90(60–180)	
Lymph-vascular invasion									
No	138	117 (84.8%)	21 (15.2%)	0(0–60)	0.049	71 (51.4%)	67 (48.6%)	90(67.5-180)	0.012
Yes	109	84 (77.1%)	25 (22.8%)	0(0–110)		67 (61.5%)	42 (38.5%)	90(0–180)	
Cirrhosis									
No	99	72 (72.7%)	27 (27.3%)	0(0–150)	0.040	53 (53.5%)	46 (46.5%)	90(50–180)	0.560
Yes	148	129 (87.2%)	19 (17.8%)	0(0–60)		85 (57.4%)	63 (42.6%)	90(22.5-180)	
AJCC clinical stage									
I	109	95(87.2%)	14 (12.8%)	0(0–60)	0.242	55 (50.5%)	54 (49.5%)	100(70–180)	0.106
II	100	74 (74.0%)	26 (26.0%)	0(0–140)		61 (61.0%)	39 (39.0%)	90(20–180)	
III	38	32 (84.2%)	6 (15.8%)	0(0–90)		22 (57.9%)	16 (42.1%)	85(0–180)	

**Table 3:** Univariate and multivariate analysis of factors associated with prognosis in HCC.

Character	*n*	Univariate analysis		Multivariate analysis^a^	
		RR (95% CI)	*P*	RR (95% CI)	*P*
Number of tumor					
Single	183	Reference		Reference	
Multiple	64	2.21 (1.56–3.14)	<0.001	1.94 (1.36–2.78)	<0.001
Tumor size					
≤5 cm	137	Reference		Reference	
>5 cm	110	1.79(1.29–2.49)	0.001	1.51(1.07–2.12)	0.018
Differentiation					
Poor	75	Reference		Reference	
Moderate/well	172	0.61(0.43–0.86)	0.005	0.79(0.54–1.14)	0.200
Lymph-vascular invasion					
No	138	Reference		Reference	
Yes	109	1.96(1.41–2.73)	<0.001	1.45(1.00–2.10)	0.050
Galectin-3					
≤120	201	Reference		Reference	
>120	46	1.64(1.11–2.42)	0.013	1.57(1.06–2.33)	0.026
Galectin-9					
≤100	138	Reference			
>100	109	1.25(0.90–1.73)	0.187		

Notes.

a Age, sex, number of tumor, tumor size, differentiation and lymph-vascular invasion were adjusted in multivariate analysis.

## Results

### Expression of Gal-3 and Gal-9 in tumor tissue and adjacent non-tumor tissue

**Figure 1:**
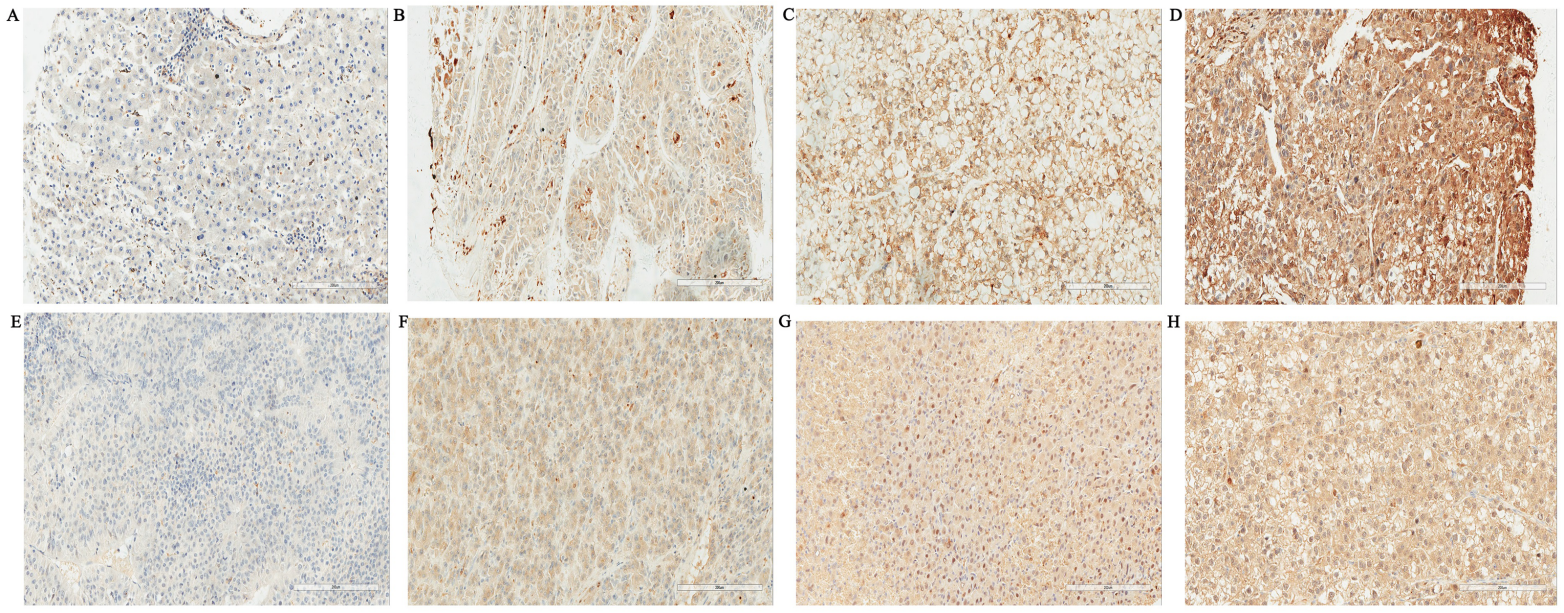
Expressions of Galectin-3 and Galectin-9 in liver tissues of tumor (SP, ×200). (A) Negative expression of Galectin-3 in tumor cells of HCC; (B) Weak Galectin-3 positive staining in tumor cells of HCC; (C) Moderate Galectin-3 positive staining in tumor cells of HCC; (D) Strong Galectin-3 positive staining in tumor cells of HCC; (E) Negative expression of Galectin-9 in tumor cells of HCC; (F) Weak Galectin-9 positive staining in tumor cells of HCC; (G) Moderate Galectin-9 positive staining in tumor cells of HCC; (H) Strong Galectin-9 positive staining in tumor cells of HCC.

Immunohistochemical analysis showed that Gal-3 and Gal-9 was diffuse stained mainly in the cytoplasm and weak stained in cell membrance and nucleus. Gal-3 and Gal-9 showed positive on tumor cells in 44.2% (121/274) and 80.7% (221/274) patients with HCC. The expression of Gal-3 and Gal-9 in liver tumor tissues was shown in Fig. 1. The positive and negative control of Gal-3 and Gal-9 were shown in Fig. S1.

**Figure 2:**
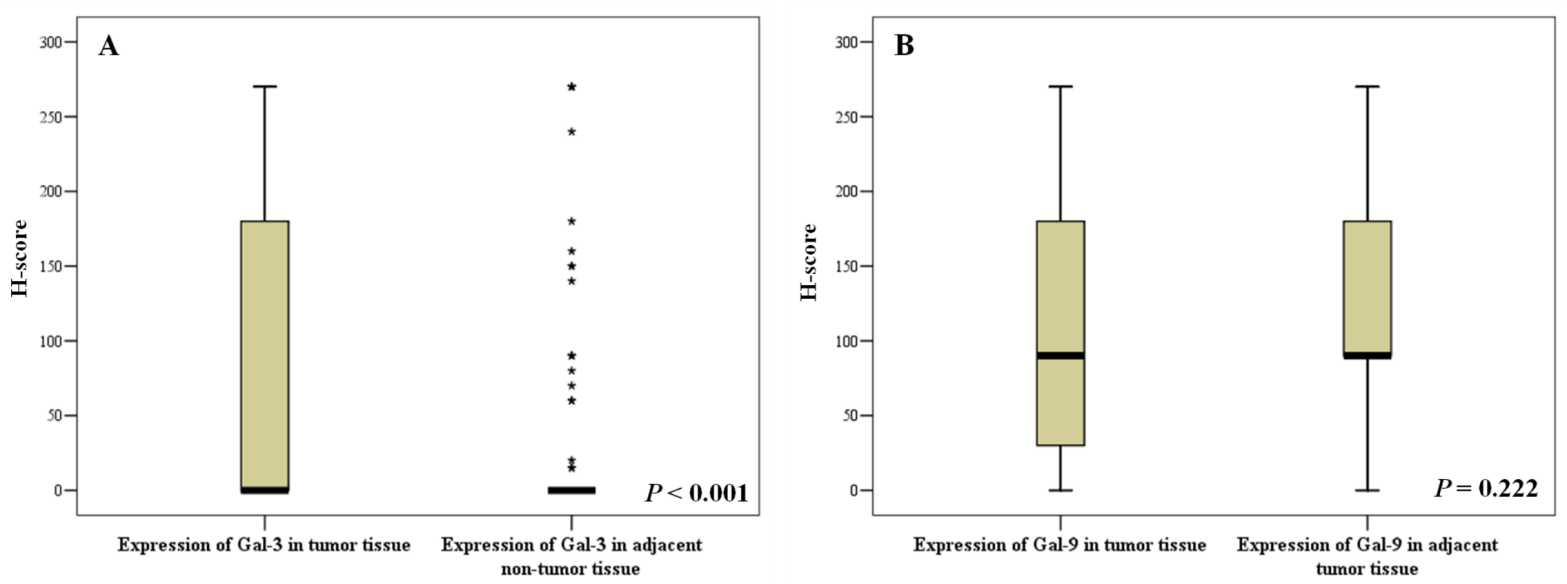
Expressions of Galectin-3 and Galectin-9 in liver tissues of tumor and adjacent non-tumor tissue. (A) Distribution of Gal-3 expression; (B) Distribution of Gal-9 expression.

Among 110 paired samples, the Gal-3 expression was significantly higher in tumor tissues than that in adjacent hepatic tissues (*P* < 0.001). However, no significant difference was found in Gal-9 expression (*P* = 0.222), (Table 1). The distribution of expression of Gal-3 and Gal-9 in tumor tissue and adjacent non-tumor tissue was shown in Fig. 2.

### Clinicopathological characters associated with the expressions of Gal-3 and Gal-9

As showed in Table 2. The expression of Gal-3 was negatively associated with the subjects who had liver cirrhosis (*P* = 0.040). The expression levels of Gal-3 was significantly higher in patients with poor histological differentiation (*P* = 0.016) and lymph-vascular invasion (*P* = 0.049). However, Gal-9 expression was significantly lower in patients with poor histological differentiation (*P* = 0.002) and lymph-vascular invasion (*P* = 0.012).

### Correlation between Gal-3 and Gal-9 expression and overall survival

**Figure 3:**
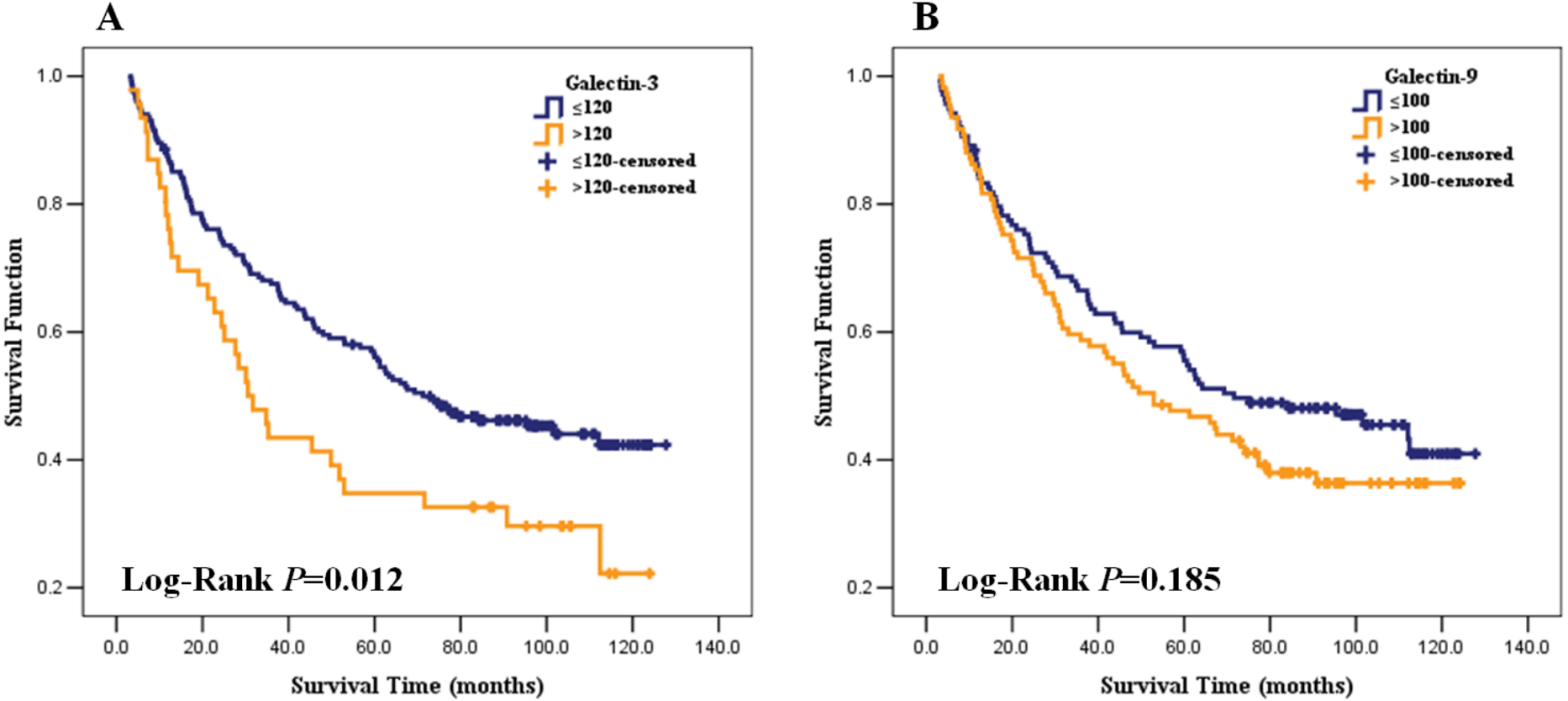
Association of Galection-3 and Galection-9 expression with overall survival in HCC patients. (A) Galection-3 expression and survival curve; (B) Galection-9 expression and survival curve.

The median follow-up time was 60 months (ranging from 3 to 128 months). During follow-up, 143 (57.9%) subjects have died of HCC, 2 (0.8%) patients died from other causes, 102 (41.3%) patients were still alive. The optimal cut-off values for Gal-3 and Gal-9 to best predict prognosis were obtained by time dependent-ROC, where the difference of the true positive (TP) and false positive (FP) was the maximum in predicting the 3-year survival. The results were H-score 120 for Gal-3 and H-score 100 for Gal-9, respectively. Kaplan–Meier survival curves of Gal-3 was plotted according to the H-score (H-score ≤ 120 and H-score >120), and the median survival time in each group were 69 months and 31months. The overall survival rate was significantly higher in Gal-3 H-score ≤ 120 group than H-score >120 group (Log-rank tests, *P* = 0.012) (Fig. 3). While median survival time in Gal-9 expression H-score ≤ 100 and H-score >100 were 71 months and 53 months. The overall survival rates of Gal-9 H-score ≤ 100 group and H-score >100 group were not significantly different (Log-rank tests, *P* = 0.185), see Fig. 3.

### Independent prognostic marker of HCC

As shown in Table 3, in univariate analysis, multiple tumor (HR = 2.21, 95% CI [1.56–3.14]), tumor size ≥ 5 cm (HR = 1.79, 95% CI [1.29–2.49]), lymph-vascular invasion (HR = 1.96, 95% CI [1.41–2.73]), AJCC clinical stage (II *vs* I: HR = 2.02, 95% CI [1. 39-2.94]; III *vs* I_1_: HR = 3.80, 95% CI [2.39–6.02]) and high Gal-3 expression (HR = 1.64, 95% CI [1.11–2.42]) were associated with poor HCC prognosis, and differentiation moderate/well (HR = 0.61, 95% CI [0.43–0.86]) was positively associated with the overall survival rate of patients with HCC, while no relationship was showed between Gal-9 expression and survival (HR = 1.25, 95% CI [0.90–1.73]). AJCC clinical stage was not brought into multiple analyses, because it was the comprehensive index of number of tumor, tumor size and lymph-vascular invasion. After adjusted by sex and age, only number of tumor (HR = 1.94, 95% CI [1.36–2.78]), tumor size ≥ 5 cm (HR = 1.51, 95% CI [1.07–2.12]), lymph-vascular invasion (HR = 1.45, 95% CI [1.00–2.10]) and Gal-3 expression (HR = 1.57, 95% CI [1.06–2.33]) remained significant associated with the prognosis in HCC patients.

## Discussion

The present study was performed on a well-characterized cohort to simultaneously evaluate the expression of Gal-3 and Gal-9 in patients with HCC. Compared with adjacent hepatic tissues, higher Gal-3 expression was observed in tumor tissues. Patients with higher Gal-3 expression had worse overall survival. Gal-3 expression in tumor tissue was found to be a potential prognostic marker in HCC.

The results of present study showed that Gal-3 expression was significantly higher in tumor tissues than that in adjacent hepatic tissues. It was consistent with previous reports (Matsuda et al., 2008; Jiang et al., 2014) and this tendency was also verified by Jiang et al. in mRNA expression by HCC lines (Jiang et al., 2014). Hsu et al. found that Gal-3 is not expressed in normal hepatocytes, while it is abundantly expressed in cirrhotic liver and hepatocellular carcinoma. It suggests that Gal-3 expression was induced in cirrhotic liver and hepatocellular carcinoma (Hsu et al., 1999). Gal-3 may take part in the formation of liver cirrhosis and hepatocellular carcinoma.

Our study confirmed that Gal-3 expression was significantly associated with lymph-vascular invasion and histological differentiation, and patients with higher Gal-3 expression had poor survival rate. It was reported by previous study (Matsuda et al., 2008). Apart from HCC, similar findings were also reported in gastric, colon and thyroid cancer (Miyazaki et al., 2002; Xu, el Naggar & Lotan, 1995; Nakamura et al., 1999). However, Jiang et al. found that Gal-3 expression had no apparent relationship with clinicopathological parameters in HCC (Jiang et al., 2014). The main reason for these differences may be a larger sample size in our study compared with the study of Jiang (*n* = 135). Another reason may be that the widely accepted semi-quantitative analysis (H-score system) was used to assess the Gal-3 expression in this study. Up-regulated Gal-3 promotes tumor growth, invasion, and migration in HCC cells in vitro (Jiang et al., 2014). Meanwhile, correlation between Gal-3 expression and micro-vessel density showed that increased Gal-3 expression in tumor cells promotes angiogenesis (Jiang et al., 2014). Collectively, this data suggested that Gal-3 may be an attractive candidate for the prediction of progression, metastasis and survival in patients with HCC.

Wang et al. reported that Gal-9 is up-regulated in human liver cancer cell lines compared to normal hepatocytes (Yang et al., 2015a). However, we found no significantly different Gal-9 expression between HCC tumor tissues and adjacent non-tumor tissues in this study. It was also different from the related study performed in other cancer, Gal-9 expression in cancer tissues of cervical squamous cell carcinoma is significant lower than that in normal epithelium (Liang et al., 2008). Previous study suggested that Gal-9 expression in tumor tissues is significantly higher than that in paired adjacent non-tumor tissues in gastric cancer (Jiang et al., 2013). The variation of Gal-9 expression appears to depend on the tissues, suggesting that the tumor specific factor may regulate the Gal-9 expression. Fewer studies focused on Gal-9 expression in HCC tumor tissues and paired normal tissues. We first found that similar expression levels of Gal-9 in HCC tumor tissues and paired adjacently non-tumor tissues. Gal-9 may not be related to hepatocarcinogenesis.

In present study, the finding that relative low expression Gal-9 was significantly correlated with histological differentiation and lymph-vascular invasion, is in accordance with results of a study reported by Zhang et al. (2012). In their study, Gal-9 expression is associated with histopathological grade, lymph node metastasis, vascular invasion and intrahepatic metastasis. The previous study confirmed that Gal-9 significantly inhibits the growth of HCC cell lines inducing apoptosis in vitro and in vivo (Fujita et al., 2015), and decreases the adhesion and invasive abilities of HepG2 cells toward extracellular matrix, and prevents the tumor metastasis (Zhang et al., 2012). However, no relationship was found between Gal-9 expression and survival time in patients with HCC based on our data. Inconsistence with our finding, under-expression of Gal-9 has been found associated with poor outcome in HCC by Sideras et al. (2017). One of the reasons for that may be in our study there are more HBV positive subjects. Enhanced expression of Gal-9 following HBV infection can interact with its ligand, T cell immunoglobulin and mucin-domain containing-3 (TIM-3), mediate T-cell senescence inducing tumor immune escape in HBV-associated HCC (Li et al., 2012; Lai et al., 2017). The other one is that the staining intensity and percentages of the stained cells were all used to assess the expression of Gal-9 in our study. The previous studies reported that Gal-9 expression is significantly associated survival time and acts as a prognostic role in various cancer, including breast cancer, gastric cancer, cervical carcinoma and malignant melanoma (Jiang et al., 2013; Zhang et al., 2012; Liang et al., 2008; Irie et al., 2005; Kageshita et al., 2002). The discrepancy from different studies may be due to tissue specificity of Gal-9 expression, genetic background, individual variation of immune state, and limited study cohort.

## Conclusions

Gal-3 expression was related to tumor oncogenesis, progression and the prognosis of HCC. Gal-3 is expected to serve as a novel prognostic marker of hepatocellular carcinoma and may have treatment value in the future. However, Gal-9 expression was only related to tumor progression but may not be a candidate for the overall survival rate of patients with HCC.

##  Supplemental Information

10.7717/peerj.9949/supp-1 Table S1 Raw Data

10.7717/peerj.9949/supp-2 Figure S1 Negative and positive control of Galectin-3 and Galectin-9 (SP, ×200) (A) Negative expression of Galectin-3 in tumor cells of HCC; (B) Positive expression of Galectin-3 in tumor cells of thyroid papillary cancer; (C) Negative expression of Galectin-9 in tumor cells of HCC; (D) Positive expression of Galectin-9 in tumor cells of gastric cancer.

## Additional Information and Declarations

### Data Availability

The following information was supplied regarding data availability: Raw data are available in the Supplemental Files.

## References

[ref-1] Bacigalupo ML, Manzi M, Rabinovich GA, Troncoso MF (2013). Hierarchical and selective roles of galectins in hepatocarcinogenesis, liver fibrosis and inflammation of hepatocellular carcinoma. World Journal of Gastroenterology.

[ref-2] Bosetti C, Turati F, La Vecchia C (2014). Hepatocellular carcinoma epidemiology. Best Practice & Research.

[ref-3] Bray F, Ferlay J, Soerjomataram I, Siegel RL, Torre LA, Jemal A (2018). Global cancer statistics 2018: GLOBOCAN estimates of incidence and mortality worldwide for 36 cancers in 185 countries. CA: A Cancer Journal for Clinicians.

[ref-4] Cai Z, Zeng Y, Xu B, Gao Y, Wang S, Zeng J, Chen L, Huang A, Liu X, Liu J (2014). Galectin-4 serves as a prognostic biomarker for the early recurrence/metastasis of hepatocellular carcinoma. Cancer Science.

[ref-5] Chen W, Zheng R, Baade PD, Zhang S, Zeng H, Bray F, Jemal A, Yu XQ, He J (2016). Cancer statistics in China, 2015. CA: A Cancer Journal for Clinicians.

[ref-6] Ebrahim AH, Alalawi Z, Mirandola L, Rakhshanda R, Dahlbeck S, Nguyen D, Jenkins M, Grizzi F, Cobos E, Figueroa JA, Chiriva-Internati M (2014). Galectins in cancer: carcinogenesis, diagnosis and therapy. Annals of Translational Medicine.

[ref-7] Fu H, Liu Y, Xu L, Liu W, Fu Q, Liu H, Zhang W, Xu J (2015). Galectin-9 predicts postoperative recurrence and survival of patients with clear-cell renal cell carcinoma. Tumour Biology.

[ref-8] Fujita K, Iwama H, Sakamoto T, Okura R, Kobayashi K, Takano J, Katsura A, Tatsuta M, Maeda E, Mimura S, Nomura T, Tani J, Miyoshi H, Morishita A, Yoneyama H, Yamana Y, Himoto T, Okano K, Suzuki Y, Niki T, Hirashima M, Masaki T (2015). Galectin-9 suppresses the growth of hepatocellular carcinoma via apoptosis in vitro and in vivo. International Journal of Oncology.

[ref-9] Hsu DK, Dowling CA, Jeng KCG, Chen J-T, Yang R-Y, Liu F-T (1999). Galectin-3 expression is induced in cirrhotic liver and hepatocellular carcinoma. International Journal of Cancer.

[ref-10] Irie A, Yamauchi A, Kontani K, Kihara M, Liu D, Shirato Y, Seki M, Nishi N, Nakamura T, Yokomise H, Hirashima M (2005). Galectin-9 as a prognostic factor with antimetastatic potential in breast cancer. Clinical Cancer Research.

[ref-11] Jiang J, Jin MS, Kong F, Cao D, Ma HX, Jia Z, Wang YP, Suo J, Cao X (2013). Decreased galectin-9 and increased Tim-3 expression are related to poor prognosis in gastric cancer. PLOS ONE.

[ref-12] Jiang SS, Weng DS, Wang QJ, Pan K, Zhang YJ, Li YQ, Li JJ, Zhao JJ, He J, Lv L, Pan QZ, Xia JC (2014). Galectin-3 is associated with a poor prognosis in primary hepatocellular carcinoma. Journal of Translational Medicine.

[ref-13] Johnston MP, Khakoo SI (2019). Immunotherapy for hepatocellular carcinoma: current and future. World Journal of Gastroenterology.

[ref-14] Kageshita T, Kashio Y, Yamauchi A, Seki M, Abedin MJ, Nishi N, Shoji H, Nakamura T, Ono T, Hirashima M (2002). Possible role of galectin-9 in cell aggregation and apoptosis of human melanoma cell lines and its clinical significance. International Journal of Cancer.

[ref-15] Lai JH, Luo SF, Wang MY, Ho LJ (2017). Translational implication of galectin-9 in the pathogenesis and treatment of viral infection. International Journal of Molecular Sciences.

[ref-16] Li H, Wu K, Tao K, Chen L, Zheng Q, Lu X, Liu J, Shi L, Liu C, Wang G, Zou W (2012). Tim-3/galectin-9 signaling pathway mediates T-cell dysfunction and predicts poor prognosis in patients with hepatitis B virus-associated hepatocellular carcinoma. Hepatology.

[ref-17] Liang M, Ueno M, Oomizu S, Arikawa T, Shinonaga R, Zhang S, Yamauchi A, Hirashima M (2008). Galectin-9 expression links to malignant potential of cervical squamous cell carcinoma. Journal of Cancer Research and Clinical Oncology.

[ref-18] Matsuda Y, Yamagiwa Y, Fukushima K, Ueno Y, Shimosegawa T (2008). Expression of galectin-3 involved in prognosis of patients with hepatocellular carcinoma. Hepatology Research.

[ref-19] McCarty Jr KS, Miller LS, Cox EB, Konrath J, McCarty Sr KS (1985). Estrogen receptor analyses. Correlation of biochemical and immunohistochemical methods using monoclonal antireceptor antibodies. Archives of Pathology and Laboratory Medicine.

[ref-20] Miyazaki J, Hokari R, Kato S, Tsuzuki Y, Kawaguchi A, Nagao S, Itoh K, Miura S (2002). Increased expression of galectin-3 in primary gastric cancer and the metastatic lymph nodes. Oncology Reports.

[ref-21] Nakahara S, Oka N, Raz A (2005). On the role of galectin-3 in cancer apoptosis. Apoptosis.

[ref-22] Nakamura M, Inufusa H, Adachi T, Aga M, Kurimoto M, Nakatani Y, Wakano T, Nakajima A, Hida JI, Miyake M, Shindo K, Yasutomi M (1999). Involvement of galectin-3 expression in colorectal cancer progression and metastasis. International Journal of Oncology.

[ref-23] Nangia-Makker P, Honjo Y, Sarvis R, Akahani S, Hogan V, Pienta KJ, Raz A (2000). Galectin-3 induces endothelial cell morphogenesis and angiogenesis. The American Journal of Pathology.

[ref-24] Radosavljevic G, Volarevic V, Jovanovic I, Milovanovic M, Pejnovic N, Arsenijevic N, Hsu D, Lukic M (2012). The roles of Galectin-3 in autoimmunity and tumor progression. Immunologic Research.

[ref-25] Sapisochin G, Bruix J (2017). Liver transplantation for hepatocellular carcinoma: outcomes and novel surgical approaches. Nature Reviews Gastroenterology & Hepatology.

[ref-26] Sideras K, Biermann K, Verheij J, Takkenberg BR, Mancham S, Hansen BE, Schutz HM, De Man RA, Sprengers D, Buschow SI, Verseput MC, Boor PP, Pan Q, Van Gulik TM, Terkivatan T, Ijzermans JN, Beuers UH, Sleijfer S, Bruno MJ, Kwekkeboom J (2017). PD-L1, Galectin-9 and CD8(+) tumor-infiltrating lymphocytes are associated with survival in hepatocellular carcinoma. Oncoimmunology.

[ref-27] Tabrizian P, Jibara G, Shrager B, Schwartz M, Roayaie S (2015). Recurrence of hepatocellular cancer after resection: patterns, treatments, and prognosis. Annals of Surgery.

[ref-28] Wu H, Chen P, Liao R, Li YW, Yi Y, Wang JX, Sun TW, Zhou J, Shi YH, Yang XR, Jin JJ, Cheng YF, Fan J, Qiu SJ (2012). Overexpression of galectin-1 is associated with poor prognosis in human hepatocellular carcinoma following resection. Journal of Gastroenterology and Hepatology.

[ref-29] Wu H, Zhang G, Li Z, Ma J, Han X, Xiang T, Jiang X (2019). Thrombospondin-4 expression as a prognostic marker in hepatocellular carcinoma. Gene.

[ref-30] Xu XC, el Naggar AK, Lotan R (1995). Differential expression of galectin-1 and galectin-3 in thyroid tumors. Potential diagnostic implications. American Journal of Pathology.

[ref-31] Yamauchi A, Kontani K, Kihara M, Nishi N, Yokomise H, Hirashima M (2006). Galectin-9, a novel prognostic factor with antimetastatic potential in breast cancer. Breast Journal.

[ref-32] Yang Q, Jiang W, Zhuang C, Geng Z, Hou C, Huang D, Hu L, Wang X (2015a). microRNA-22 downregulation of galectin-9 influences lymphocyte apoptosis and tumor cell proliferation in liver cancer. Oncology Reports.

[ref-33] Yang XD, Pan LH, Wang L, Ke Y, Cao J, Yang C, Zhong JH, Luo W, Guo J, Li LQ (2015b). Systematic review of single large and/or multinodular hepatocellular carcinoma: surgical resection improves survival. Asian Pacific Journal of Cancer Prevention.

[ref-34] Zeng H, Chen W, Zheng R, Zhang S, Ji JS, Zou X, Xia C, Sun K, Yang Z, Li H, Wang N, Han R, Liu S, Mu H, He Y, Xu Y, Fu Z, Zhou Y, Jiang J, Yang Y, Chen J, Wei K, Fan D, Wang J, Fu F, Zhao D, Song G, Jiang C, Zhou X, Gu X, Jin F, Li Q, Li Y, Wu T, Yan C, Dong J, Hua Z, Baade P, Bray F, Jemal A, Yu XQ, He J (2018). Changing cancer survival in China during 2003–15: a pooled analysis of 17 population-based cancer registries. Lancet Glob Health.

[ref-35] Zhang ZY, Dong JH, Chen YW, Wang XQ, Li CH, Wang J, Wang GQ, Li HL, Wang XD (2012). Galectin-9 acts as a prognostic factor with antimetastatic potential in hepatocellular carcinoma. Asian Pacific Journal of Cancer Prevention.

