# Supplementary material for: Galectin-3 not Galectin-9 as a candidate prognosis marker for hepatocellular carcinoma
Source: PeerJ. 2020 Sep 16;8:e9949. doi: 10.7717/peerj.9949 (PMC7501799; doi:10.7717/peerj.9949)
Supplement: Table S1 [file peerj-08-9949-s001.pdf]

No.	sex	age	HBV	HCV	Differentiation	number of tumor	Tumor Size	Lymph-vascular invasion	AJCC	Cirrhosis	Gal-3	Gal-9	Survival time	endpoints
279,269	1	69	negative	positive	moderate	single	4.0	no	I	Yes	0	90	44.0	death
281,455	1	67	positive	negative	moderate	single	6.0	no	I	No	0	0	127.8	survival
288,531	1	63	negative	positive	moderate	single	7.0	no	I	Yes	270	180	7.3	death
289,183	2	60	positive	negative	poor	single	5.0	no	II	Yes	0	180	124.2	survival
289,890	1	60	positive	negative	well	single	3.5	no	I	Yes	210	90	123.9	survival
290,074	1	46	positive	negative	well	single	6.0	Yes	II	Yes	0	180	123.9	survival
290,474	1	55	negative	negative	well	single	6.0	no	I	No	0	270	123.9	survival
291,973	1	63	positive	negative	well	single	4.5	no	I	Yes	0	90	123.2	survival
292,429	2	61	positive	negative	well	single	3.4	no	I	Yes	0	90	123.2	survival
292,802	1	53	negative	positive	well	single	2.7	no	I	Yes	0	180	46.1	death
292,961	1	46	positive	negative	moderate	single	5.5	no	I	Yes	0	0	8.2	death
293,346	2	58	negative	positive	well	single	3.5	no	I	No	0	180	122.7	survival
294,775	1	34	positive	negative	moderate	single	1.0	no	II	Yes	0	0	122.2	survival
295,048	1	27	positive	negative	poor	multiple	7.0	Yes	III	No	15	80	3.8	death
295,954	2	60	positive	negative	poor	single	4.0	Yes	II	No	240	180	5.6	death
296,313	1	53	positive	negative	moderate	single	8.0	Yes	II	No	270	180	27.7	death
296,991	1	44	positive	negative	moderate	single	2.0	no	I	Yes	0	60	14.9	death
297,396	1	71	negative	positive	moderate	multiple	7.0	Yes	III	Yes	30	0	121.1	survival
297,621	1	61	negative	negative	poor	single	7.0	Yes	II	Yes	20	0	95.3	death
297,979	1	60	positive	negative	well	single	4.0	no	I	Yes	80	90	121.2	survival
298,248	2	56	positive	negative	moderate	multiple	2.7	Yes	II	Yes	20	180	61.2	death
298,430	1	55	positive	negative	moderate	single	2.0	no	II	Yes	80	180	30.7	death
298,650	1	50	positive	negative	moderate	single	4.5	no	I	Yes	0	180	24.9	death
298,671	1	71	positive	negative	well	single	6.5	no	I	No	270	0	51.8	death
300,144	1	58	negative	negative	moderate	single	5.0	no	I	No	60	270	73.1	death
301,333	1	46	negative	negative	moderate	single	8.0	no	I	Yes	0	0	120.0	survival
301,734	2	54	positive	negative	poor	single	6.5	no	I	Yes	0	180	4.6	death
302,873	2	41	positive	negative	poor	single	2.0	no	I	Yes	0	0	82.7	survival
303,670	1	62	positive	negative	poor	single	13.5	Yes	II	Yes	0	80	119.1	survival
304,284	1	59	negative	positive	moderate	single	9.0	no	I	Yes	120	90	75.1	death
306,391	1	64	positive	negative	poor	multiple	3.5	no	I	Yes	40	160	17.7	death
307,110	1	44	positive	negative	well	single	6.5	no	I	Yes	10	70	117.8	survival
309,771	1	57	positive	negative	well	single	2.5	no	I	Yes	0	180	38.0	death
309,818	1	57	positive	negative	poor	multiple	8.0	Yes	II	Yes	0	0	116.4	survival
310,384	1	72	negative	positive	moderate	single	5.0	Yes	II	Yes	210	90	49.9	death
310,634	2	49	positive	negative	poor	multiple	4.5	Yes	II	Yes	90	270	116.3	survival
310,977	1	55	positive	negative	poor	single	3.5	Yes	II	Yes	180	0	116.0	survival
311,748	1	60	positive	negative	well	single	6.0	Yes	II	No	0	270	16.8	death
311,749	1	47	positive	negative	moderate	single	2.5	no	I	Yes	0	140	115.7	survival
311,784	1	62	positive	negative	moderate	multiple	11.5	Yes	III	No	0	60	7.7	death

312,622	1	52	positive	positive	poor	multiple	5.0	Yes	III	No	0	0	115.4	survival
314,019	1	40	positive	negative	well	single	3.0	Yes	II	Yes	0	180	12.9	death
314,333	1	55	positive	negative	moderate	multiple	3.5	Yes	II	No	120	70	11.2	lose to follow up
314,513	2	64	positive	negative	poor	single	6.5	Yes	II	No	150	0	114.6	survival
314,523	2	56	positive	negative	moderate	single	1.0	Yes	II	Yes	90	0	112.1	death
314,637	2	37	positive	negative	moderate	single	2.5	no	I	Yes	60	180	114.5	survival
315,125	1	46	negative	positive	poor	multiple	6.0	Yes	III	Yes	0	90	59.8	death
315,329	1	47	positive	negative	well	single	2.5	no	I	Yes	0	180	114.5	survival
315,717	1	55	positive	negative	moderate	single	5.0	no	I	Yes	240	90	24.4	death
315,805	2	44	negative	positive	moderate	single	8.0	Yes	II	Yes	0	90	101.5	death
315,843	2	69	negative	positive	moderate	single	1.3	no	I	Yes	0	0	114.0	survival
315,857	1	61	negative	positive	moderate	single	7.0	no	III	Yes	0	180	26.6	death
316,109	1	45	positive	negative	moderate	single	4.5	no	I	Yes	150	90	112.4	death
316,490	2	49	positive	negative	poor	single	5.0	Yes	II	Yes	0	180	114.0	survival
317,211	2	56	positive	negative	moderate	multiple	14.0	Yes	III	Yes	90	120	3.8	death
317,646	1	59	positive	negative	moderate	multiple	8.0	no	III	Yes	180	0	30.6	death
317,710	2	60	negative	negative	poor	single	5.5	no	I	Yes	60	0	62.6	death
317,981	1	61	positive	negative	moderate	single	8.0	Yes	II	No	0	90	113.2	survival
318,277	2	70	negative	negative	well	single	4.5	no	I	Yes	0	180	77.2	death
318,365	1	38	positive	negative	poor	multiple	5.0	no	II	Yes	0	0	60.0	death
318,795	1	35	positive	negative	poor	single	6.0	Yes	II	Yes	0	90	5.7	death
318,929	1	56	positive	negative	moderate	single	6.5	no	I	No	0	70	113.1	survival
319,124	1	51	positive	negative	moderate	single	6.5	no	II	No	0	60	112.8	survival
319,329	1	46	positive	negative	poor	single	14.0	Yes	II	No	210	180	11.4	death
319,883	1	60	positive	negative	moderate	single	3.0	no	I	Yes	0	180	112.2	survival
319,916	1	65	negative	positive	poor	single	12.0	Yes	II	Yes	0	0	3.4	death
320,265	1	59	negative	positive	moderate	single	2.0	Yes	II	Yes	0	210	48.0	death
320,988	1	69	negative	positive	moderate	single	4.5	no	I	No	0	60	45.6	death
321,339	1	33	positive	negative	poor	single	21.0	Yes	III	Yes	0	0	7.3	death
322,047	1	51	positive	negative	poor	single	8.5	Yes	II	Yes	0	30	17.3	death
322,286	1	47	positive	negative	poor	multiple	2.6	Yes	II	No	210	180	12.5	death
323,136	1	39	positive	negative	poor	single	12.0	Yes	III	No	0	80	111.2	survival
323,500	1	54	positive	negative	moderate	single	8.5	no	I	Yes	0	180	29.5	death
324,028	1	69	positive	negative	moderate	multiple	11.0	no	III	Yes	0	0	12.0	death
324,692	1	58	negative	negative	well	single	3.0	no	I	Yes	0	0	110.7	survival
324,967	1	54	positive	negative	well	single	2.5	no	II	Yes	0	270	41.4	death
325,111	1	66	negative	negative	well	single	8.0	no	I	No	160	90	71.6	death
325,825	1	55	positive	negative	moderate	multiple	4.0	no	II	No	210	90	34.8	death
326,399	1	48	positive	negative	moderate	multiple	7.0	Yes	III	No	0	80	8.3	death
327,133	1	44	positive	negative	moderate	multiple	3.5	Yes	II	No	0	0	3.4	death
327,769	1	66	positive	negative	moderate	multiple	11.0	Yes	III	No	0	180	9.1	death

328,575	1	60	positive	negative	poor	multiple	8.0	no	III	No	270	270	10.1	death
328,755	2	44	positive	negative	moderate	single	5.5	Yes	II	No	240	0	6.8	death
329,608	2	52	positive	negative	well	single	3.5	no	I	No	100	90	109.0	survival
330,498	1	41	positive	negative	moderate	single	2.5	Yes	II	No	0	70	108.7	survival
331,583	1	61	negative	positive	moderate	single	3.5	no	I	Yes	0	180	108.3	survival
331,934	1	66	negative	positive	poor	single	5.0	Yes	II	No	100	180	108.2	survival
333,329	1	49	positive	negative	poor	multiple	12.0	no	III	Yes	0	270	5.3	death
335,610	1	54	positive	negative	well	single	12.0	no	I	No	0	90	43.7	death
335,713	1	51	positive	negative	moderate	multiple	6.0	Yes	III	No	0	270	16.3	death
339,666	2	47	positive	negative	moderate	single	5.0	Yes	II	Yes	270	90	105.7	survival
340,364	1	56	negative	negative	poor	multiple	7.5	Yes	III	No	30	40	9.7	death
340,819	1	48	positive	negative	moderate	single	5.0	no	I	No	150	270	105.4	survival
341,187	1	56	negative	negative	well	single	6.0	no	I	No	0	270	49.5	death
343,633	1	55	negative	positive	moderate	single	4.6	Yes	II	Yes	0	0	11.5	death
344,657	1	53	negative	negative	moderate	multiple	8.5	Yes	III	No	180	0	11.5	death
344,950	1	41	positive	negative	poor	single	2.3	no	I	Yes	270	90	103.8	survival
345,936	1	57	positive	negative	poor	single	7.0	no	I	Yes	180	270	103.4	survival
346,079	1	38	positive	negative	moderate	single	10.5	Yes	II	Yes	0	240	35.9	death
346,616	1	38	negative	negative	well	single	4.7	no	I	No	80	90	69.3	death
347,562	1	40	negative	negative	moderate	single	2.5	Yes	II	No	270	0	30.1	death
348,523	2	38	negative	negative	moderate	multiple	6.0	no	III	No	0	90	102.7	survival
349,137	1	62	negative	negative	well	single	9.0	Yes	II	No	0	50	102.4	survival
349,251	2	66	negative	positive	well	single	6.0	no	I	No	140	180	90.8	death
350,394	1	48	positive	negative	well	single	6.0	no	I	No	30	0	102.2	survival
351,689	1	45	positive	negative	moderate	multiple	3.0	no	II	No	270	270	25.1	death
352,463	1	55	positive	negative	poor	single	3.0	Yes	II	Yes	0	90	15.6	death
352,708	1	53	positive	negative	moderate	single	8.0	Yes	II	No	210	50	22.8	death
353,066	1	54	negative	positive	well	single	4.0	no	I	No	0	90	101.3	survival
353,404	1	61	positive	negative	moderate	multiple	10.0	Yes	III	Yes	0	90	5.2	death
353,556	1	43	positive	negative	moderate	single	4.5	Yes	II	No	0	0	100.8	survival
353,604	1	72	positive	negative	moderate	multiple	8.0	Yes	III	No	20	180	15.1	death
355,744	1	58	positive	positive	moderate	single	3.0	no	I	Yes	60	180	93.6	survival
356,155	1	75	negative	negative	moderate	single	5.0	no	I	No	0	0	100.1	survival
356,871	1	45	positive	negative	moderate	multiple	9.0	Yes	III	Yes	0	270	9.0	death
357,134	1	39	positive	negative	well	single	14.0	Yes	III	No	210	180	7.3	death
357,236	1	61	positive	negative	well	multiple	2.5	no	II	Yes	180	80	45.4	death
357,466	1	74	negative	positive	poor	single	2.0	Yes	II	Yes	0	40	39.3	death
358,610	1	58	positive	negative	poor	single	1.6	no	I	Yes	0	0	53.0	death
358,718	1	48	negative	negative	poor	single	3.5	Yes	II	No	0	90	99.2	survival
359,177	1	56	negative	negative	well	multiple	4.5	no	II	No	0	90	37.5	death
360,445	2	50	positive	negative	moderate	multiple	5.8	Yes	III	Yes	15	0	98.7	survival

361,028	1	40	positive	negative	poor	single	6.5	Yes	II	Yes	0	0	12.8	death
362,342	1	49	positive	negative	moderate	multiple	3.5	no	II	No	270	90	98.4	survival
362,873	2	57	positive	negative	poor	multiple	5.5	Yes	III	Yes	0	0	4.3	death
362,976	2	69	negative	positive	well	single	5.5	no	I	Yes	0	90	17.5	death
363,167	2	56	positive	negative	moderate	multiple	4.0	Yes	II	Yes	150	90	14.4	death
363,377	1	65	positive	negative	poor	single	12.0	Yes	III	Yes	90	140	3.6	death
363,977	1	83	negative	negative	well	multiple	3.0	no	I	No	270	270	5.0	death
364,111	1	54	negative	positive	moderate	multiple	1.6	Yes	II	Yes	0	70	97.9	survival
364,382	1	48	positive	negative	moderate	single	3.6	no	I	Yes	0	80	97.6	survival
364,502	1	55	negative	positive	moderate	single	4.0	no	I	Yes	0	0	97.6	survival
364,725	2	65	negative	positive	poor	multiple	8.5	Yes	III	Yes	0	0	97.5	survival
365,173	2	31	positive	negative	well	single	7.5	no	I	Yes	120	90	97.4	survival
365,220	1	57	positive	negative	moderate	single	10.0	no	I	Yes	0	90	97.6	survival
365,498	2	65	negative	positive	well	single	3.0	no	I	Yes	0	90	97.3	survival
366,266	1	57	positive	negative	poor	multiple	5.6	Yes	III	Yes	90	30	97.1	survival
366,858	1	55	negative	negative	well	single	2.8	no	I	No	0	180	97.0	survival
367,331	2	23	positive	negative	moderate	single	5.0	no	I	Yes	120	0	96.9	survival
368,084	1	48	negative	negative	poor	single	16.0	Yes	II	No	270	0	19.1	death
368,427	1	40	positive	negative	poor	multiple	7.0	Yes	III	No	270	0	2.8	death
369,638	1	41	negative	negative	well	single	1.5	no	I	No	0	270	96.3	survival
369,819	1	35	positive	negative	well	single	4.0	Yes	II	No	0	0	59.2	death
370,433	1	56	positive	negative	moderate	single	1.5	no	I	No	0	120	9.2	death
371,471	1	67	negative	negative	moderate	single	5.4	no	I	No	0	180	95.7	survival
371,931	1	49	positive	negative	moderate	single	4.0	Yes	II	Yes	0	140	79.7	death
372,587	1	58	positive	negative	moderate	single	7.0	Yes	II	No	180	0	95.3	survival
373,063	1	60	negative	positive	well	single	3.5	no	I	No	0	120	95.2	survival
373,336	1	59	positive	negative	poor	single	3.7	no	I	No	0	30	64.2	death
373,499	2	62	positive	negative	moderate	multiple	4.0	no	II	Yes	0	180	24.6	death
373,721	1	54	positive	negative	poor	multiple	3.0	Yes	II	No	90	270	77.3	death
374,003	1	36	positive	negative	poor	multiple	2.3	no	II	Yes	240	270	31.7	death
374,342	1	67	negative	positive	moderate	multiple	5.8	no	II	No	0	0	84.2	death
374,532	1	48	positive	negative	poor	single	9.0	Yes	II	No	140	180	21.2	death
375,270	1	61	positive	negative	moderate	multiple	14.0	Yes	III	No	0	270	5.8	death
378,919	1	58	positive	negative	poor	multiple	7.5	Yes	III	Yes	0	140	67.6	death
379,596	1	71	negative	positive	moderate	multiple	10.0	no	II	No	0	40	4.5	death
379,748	1	59	negative	positive	moderate	single	3.0	no	I	Yes	0	270	93.0	survival
379,899	1	54	positive	negative	moderate	multiple	2.5	no	I	Yes	120	0	93.3	survival
380,032	1	60	negative	negative	moderate	single	12.0	no	I	No	0	0	93.1	survival
380,379	2	42	positive	negative	moderate	single	3.0	no	I	Yes	0	100	92.9	survival
380,445	1	43	positive	negative	moderate	single	3.5	no	I	Yes	0	80	93.1	survival
380,601	1	48	positive	negative	moderate	single	5.0	Yes	II	Yes	140	180	52.9	death

380,667	1	73	negative	positive	poor	single	3.8	Yes	II	Yes	0	180	92.9	survival
380,813	1	45	positive	negative	moderate	single	4.5	Yes	II	Yes	0	180	74.1	death
382,567	1	64	negative	negative	moderate	multiple	3.0	no	II	Yes	0	270	19.7	death
382,715	2	43	positive	negative	poor	multiple	4.5	Yes	II	Yes	30	90	33.9	death
383,247	1	48	positive	negative	poor	multiple	4.5	Yes	II	Yes	60	90	19.8	death
383,584	2	49	positive	negative	moderate	multiple	7.8	Yes	III	Yes	90	180	31.1	death
384,365	1	56	positive	negative	well	single	2.0	no	I	Yes	0	60	16.1	death
385,001	1	59	positive	negative	moderate	single	7.0	no	I	Yes	0	270	20.4	death
385,725	1	50	negative	negative	poor	single	22.0	Yes	III	Yes	60	120	12.7	death
386,638	1	60	negative	negative	moderate	single	2.5	no	I	No	0	180	91.2	survival
386,916	1	55	negative	positive	moderate	single	1.5	Yes	II	Yes	0	0	91.0	survival
387,002	2	40	positive	negative	moderate	single	3.7	no	I	Yes	0	180	46.9	death
387,273	2	58	negative	positive	moderate	single	5.0	no	I	Yes	0	180	71.2	death
388,774	1	35	positive	negative	poor	single	1.0	no	I	Yes	0	0	90.5	survival
391,919	1	55	positive	negative	moderate	multiple	2.5	no	I	Yes	0	270	15.7	death
393,445	1	38	positive	negative	poor	multiple	5.0	Yes	II	No	180	0	12.0	death
393,651	1	37	positive	negative	moderate	multiple	2.5	no	II	Yes	0	90	89.2	survival
393,848	2	55	positive	negative	moderate	single	2.5	Yes	II	Yes	0	180	89.1	survival
394,780	1	56	positive	negative	poor	single	4.8	Yes	II	No	0	140	27.3	death
395,214	1	57	negative	negative	moderate	multiple	9.0	Yes	III	No	0	180	88.5	survival
398,418	2	55	positive	negative	poor	single	8.5	Yes	II	Yes	150	20	35.3	death
399,791	1	60	positive	negative	poor	single	7.8	Yes	II	Yes	270	180	12.8	death
399,983	1	60	negative	positive	poor	single	7.5	Yes	II	No	270	90	87.4	survival
400,898	1	62	positive	negative	well	single	5.5	no	I	No	0	180	33.0	death
401,885	2	65	negative	positive	poor	single	3.5	no	I	Yes	60	150	17.3	death
402,678	2	48	negative	negative	moderate	single	6.5	no	I	No	270	270	86.8	survival
405,210	1	45	positive	negative	poor	single	7.5	Yes	II	Yes	0	60	20.9	death
405,389	1	43	negative	negative	moderate	single	8.5	Yes	II	No	0	180	10.5	death
406,412	1	48	positive	negative	poor	single	2.7	Yes	II	Yes	80	60	37.8	death
408,740	1	56	negative	negative	well	multiple	4.0	no	II	No	0	60	85.4	survival
409,008	1	53	positive	negative	moderate	single	4.5	no	I	No	80	240	31.2	death
409,179	1	62	positive	negative	moderate	single	10.5	no	I	Yes	0	270	42.0	death
409,501	1	52	positive	negative	moderate	single	1.5	no	I	Yes	0	270	85.2	survival
409,699	1	69	positive	negative	moderate	single	5.2	Yes	II	No	180	90	9.7	death
409,833	2	51	positive	negative	well	multiple	9.5	no	III	No	0	180	20.2	death
411,016	1	60	positive	negative	poor	single	4.5	no	I	No	60	0	84.7	survival
411,295	2	66	positive	negative	poor	multiple	3.0	Yes	II	Yes	0	90	12.2	death
411,536	2	70	negative	positive	moderate	single	2.0	no	I	Yes	0	140	84.5	survival
411,655	1	52	positive	negative	poor	single	9.5	Yes	II	Yes	0	0	24.1	death
411,702	1	60	negative	negative	moderate	single	2.5	no	I	No	0	90	84.5	survival
411,775	1	66	positive	negative	moderate	multiple	4.0	no	II	No	0	140	84.4	survival

413,494	2	68	negative	positive	moderate	single	4.5	no	I	Yes	0	180	83.8	survival
413,564	1	57	negative	positive	moderate	multiple	3.5	no	II	No	0	180	45.8	death
415,071	1	75	negative	positive	moderate	single	5.0	no	I	No	0	80	62.4	death
415,274	2	36	positive	negative	poor	single	9.5	Yes	II	No	0	180	8.5	death
415,407	1	38	positive	negative	moderate	single	6.5	no	I	Yes	180	270	83.1	survival
416,198	1	59	positive	negative	moderate	single	5.0	no	I	Yes	0	180	82.8	survival
416,213	1	75	positive	negative	moderate	single	6.0	no	I	Yes	210	270	82.9	survival
416,802	1	62	negative	positive	moderate	single	4.5	no	I	Yes	0	210	54.9	lose to follow up
416,806	1	55	positive	negative	moderate	single	6.2	Yes	II	Yes	0	270	82.8	survival
417,065	1	56	positive	negative	poor	single	7.0	Yes	II	Yes	0	180	43.5	death
417,799	1	54	negative	negative	poor	single	12.0	Yes	II	Yes	0	180	15.8	death
418,845	2	52	positive	negative	moderate	single	9.0	Yes	II	Yes	40	0	37.6	death
419,549	1	38	positive	negative	moderate	single	1.0	no	I	Yes	60	180	66.0	death
419,870	2	63	positive	negative	poor	single	6.5	no	I	Yes	0	90	11.7	death
420,155	1	65	negative	negative	poor	single	3.5	no	I	No	90	80	60.7	death
422,430	1	56	positive	negative	moderate	single	2.2	no	I	Yes	0	90	80.4	survival
423,697	2	56	negative	positive	poor	single	4.5	no	I	Yes	0	70	63.3	death
423,930	1	53	positive	negative	moderate	single	3.0	no	I	Yes	0	160	79.9	survival
424,781	1	51	positive	negative	poor	single	3.0	Yes	II	Yes	100	20	79.7	survival
426,652	1	54	positive	negative	moderate	single	4.8	Yes	II	No	0	210	78.9	survival
427,031	1	45	positive	negative	poor	single	8.0	Yes	II	Yes	120	0	29.4	death
427,148	1	63	negative	positive	moderate	single	9.0	no	I	No	0	120	78.7	survival
427,149	1	56	negative	negative	moderate	single	5.5	Yes	II	No	0	180	78.8	survival
428,037	1	48	positive	negative	moderate	multiple	12.0	Yes	III	Yes	150	20	28.4	death
428,265	1	36	negative	negative	moderate	multiple	7.5	no	III	No	15	40	38.3	death
428,508	1	60	negative	positive	moderate	single	2.0	Yes	II	Yes	0	180	29.9	death
429,160	1	68	positive	negative	moderate	single	6.0	Yes	II	No	0	60	78.0	survival
429,312	1	55	positive	negative	moderate	single	4.5	Yes	II	Yes	0	20	77.9	survival
433,275	2	47	positive	negative	poor	single	3.2	no	I	No	120	160	52.9	death
433,293	1	54	positive	negative	moderate	single	4.0	no	I	Yes	0	180	76.5	survival
433,408	1	51	positive	negative	well	single	3.0	no	I	No	0	180	56.7	death
433,996	1	50	positive	negative	poor	single	2.3	no	I	Yes	0	50	61.2	death
434,753	1	65	positive	negative	moderate	single	6.7	no	I	No	0	160	67.1	death
435,165	1	44	positive	negative	moderate	single	4.5	no	I	Yes	0	80	75.6	survival
435,203	2	56	positive	negative	poor	single	2.7	no	I	Yes	0	0	75.6	survival
435,271	1	70	negative	positive	poor	multiple	4.0	Yes	II	Yes	60	0	16.2	death
435,712	1	43	positive	negative	poor	single	4.8	no	I	No	60	0	75.4	survival
435,897	1	71	positive	negative	moderate	single	5.0	Yes	II	Yes	0	60	11.2	death
437,496	1	58	positive	negative	moderate	single	6.7	Yes	III	Yes	0	60	27.7	death
438,316	2	41	negative	negative	well	single	6.0	no	I	No	0	180	74.7	survival
439,550	2	64	negative	negative	moderate	single	2.2	no	I	Yes	60	60	24.0	death

443,202	1	49	positive	negative	moderate	single	4.0	no	II	Yes	0	70	23.8	death
443,524	1	66	positive	negative	well	single	7.0	no	I	No	0	270	72.9	survival
