# Supplementary material for: Galectin-3 not Galectin-9 as a candidate prognosis marker for hepatocellular carcinoma
Source: PeerJ. 2020 Sep 16;8:e9949. doi: 10.7717/peerj.9949 (PMC7501799; doi:10.7717/peerj.9949)
Supplement: Figure S1 — (A) Negative expression of Galectin-3 in tumor cells of HCC; (B) Positive expression of Galectin-3 in tumor cells of thyroid papillary cancer; (C) Negative expression of Galectin-9 in tumor cells of HCC; (D) Positive expression of Galectin-9 in tumor cells of gastric cancer. [file peerj-08-9949-s002.pdf]

Supplementary figure: Negative and positive control of Galectin-3 and Galectin-9 (SP, ×200).

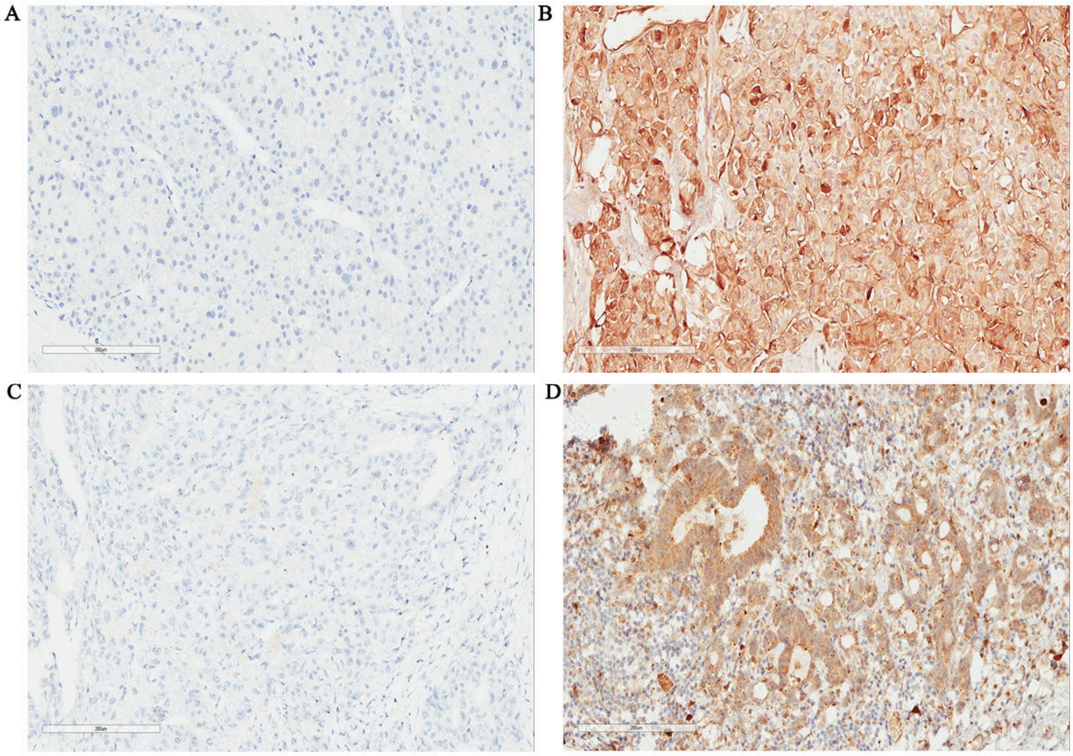

(A) Negative expression of Galectin-3 in tumor cells of HCC; (B) Positive expression of Galectin-3 in tumor cells of thyroid papillary cancer; (C) Negative expression of Galectin-9 in tumor cells of HCC; (D) Positive expression of Galectin-9 in tumor cells of gastric cancer;
